# Analysis of barriers associated with emergency medical service activation in patients with acute stroke and acute myocardial infarction from Zhongjiang County of Sichuan Province in China

**DOI:** 10.1186/s12873-024-01035-5

**Published:** 2024-07-09

**Authors:** Chengcheng He, Yingchun Zhang, Meimei Tang, Xiaohua Ai, Mingxiang Tang, Cheng Tang, Li Li, Wenjin Huang, Xin You, Dewen Zhou, Jiming Zhou, Yan Shi, Min Luo

**Affiliations:** 1 Department of Emergency Medicine, People’s hospital of Zhongjiang, Deyang, Sichuan China; 2 Department of Neurology, People’s hospital of Zhongjiang, Deyang, Sichuan China

**Keywords:** Emergency medical service, Acute stroke, Acute myocardial infarction, Barriers self-transportation

## Abstract

**Objectives:**

The purpose of this study was to investigate the preferred modes of transportation to the hospital among patients with acute stroke and acute myocardial infarction (AMI), as well as to identify the factors that influence the utilization of ambulances.

**Methods:**

We conducted a cross-sectional study, including patients who were diagnosed with acute stroke and AMI, at the people’s hospital of Zhongjiang, from September 30th, 2022 to August 30th, 2023. All patients were divided into emergency medical service (EMS)-activation group and self-transportation group. Chi-square and t-tests were utilized to discern differences between groups at baseline. To screen relevant variables, we employed the Least Absolute Shrinkage and Selection Operator (LASSO) regression analysis using R package glmnet. Subsequently, we performed a logistic regression analysis to identify predictors of EMS activation according the results of LASSO regression.

**Results:**

we collected 929 valid questionnaires. 26.16% of the patients required the services of EMS. 90.9% of individuals have not received any formal first aid education. 42.1% of them reported that they had no understanding of cardiovascular and cerebrovascular diseases. Diagnosed as AMI (OR 0.22, 95%CI 0.06 to 0.88) or acute cerebral infarction (OR 0.26, 0.10 to 0.68), the distance between the patient and the nearest 120 network hospital when the patient had these symptoms (OR 0.97, 0.94 to 0.99), the patient’s son or daughter was there when the patient was symptomatic (OR 0.58, 0.37 to 0.94), the patient (OR 0.19, 0.05 to 0.72) and the patient’s partner (wife or husband) (OR 0.36, 0.16 to 0.85) had decided that the patient needed further medical help, Among patients who did not seek immediate help after symptom onset, thinking that the symptoms will disappear spontaneously (OR 0.34, 0.13 to 0.92) or not wanting to disturb others (OR 0.06, 0.01 to 0.66) or believing that they are not important symptoms (OR 0.15, 0.05 to 0.42) were factors independently associated with less ambulance use. Age (OR 1.02, 1.00 to 1.04), Stroke patients have experienced symptoms of disturbance of consciousness or convulsions (OR 2.99, 1.72 to 5.2) were independent factors associated with increased ambulance use.

**Conclusion:**

There is still ambulance underutilization among patients with acute stroke and AMI in county territory of China. Moreover, it is needed to raise the level of first aid education and awareness about EMS. Additionally, private clinic doctors and the public should gain adequate understanding of the severity of acute stroke and AMI, as well as their common symptoms, the crucial importance of prompt medical intervention. Finally, we propose that all township hospitals should be integrated into the 120 emergency networks and equipped with emergency first aid capabilities, pre-hospital care, and transportation abilities.

**Supplementary Information:**

The online version contains supplementary material available at 10.1186/s12873-024-01035-5.

## Introduction

Acute stroke and acute myocardial infarction (AMI) pose a significant threat to the health of the Chinese population [1, 2]. According to the 2019 Global Burden of Disease study, China experienced 3.94 million new cases of stroke in 2019, with 28.76 million existing cases. Of these cases, 2.19 million resulted in death, with 82.6% being ischemic strokes [2]. Stroke exhibits high mortality and disability rates, serving as a major cause of death and disability among Chinese adults [2]. For acute ischemic stroke, prompt interventions such as intravenous thrombolysis and endovascular treatment administered within the critical time window can effectively dissolve blood clots and restore blood flow [3]. This timely approach significantly improves clinical outcomes, reduces mortality rates, and minimizes disability [3]. Similarly, AMI has a high incidence and mortality rate worldwide [4].In China, there is a high incidence rate of coronary artery disease(CAD), and the mortality rate of AMI has shown a rapid and continuous upward trend from 2012 to 2020. Specifically, the AMI mortality rate increased from 68.62 to 135.88 per 100,000 rural population, and from 93.17 to 126.91 per 100,000 urban population annually [5]. Early reperfusion therapy, including intravenous thrombolysis and percutaneous coronary intervention (PCI), is crucial for managing AMI [1, 4].

Emergency Medical Service (EMS) play a crucial role in swiftly transporting patients and providing initial treatment, and early activation of EMS is vital for promptly diagnosing and treating acute stroke and AMI patients [6]. For patients experiencing acute stroke and AMI, the timely activation of EMS is paramount. The direct correlation between prompt EMS activation and improved clinical outcomes is crucial in ensuring optimal patient care and recovery [6, 7]. However, in China, especially in rural or county-level areas, the utilization of ambulances for acute ischemic stroke and AMI patients remains low [6]. Some studies have explored factors influencing the non-use of ambulances by patients with acute coronary syndrome [6, 8–11] and the pre-hospital delay in stroke patients [7]. However, data regarding reasons for the delayed initiation of EMS in acute stroke and AMI patients in county-level areas are lacking.

This study aims to analyze the barriers preventing the initiation of EMSS in patients with acute stroke and AMI, which could contribute to targeted solutions and improved time management for these conditions. This would subsequently enhance patient outcomes and provide greater benefits to affected individuals. Moreover, the research findings can serve as valuable references for county-level areas in China, benefiting a diverse populace and potentially contributing to socioeconomic improvements.

## Methods

### Study type and population

We conducted a prospective, single-center, cross-sectional study, including the patients who were diagnosed with acute stroke and AMI, at the people’s hospital of Zhongjiang, a tertiary hospital in Zhongjiang County of Sichuan Province, from September 30, 2022, to August 30, 2023. All the patients were onseted in the territory of Zhongjiang County. Some came to our hospital by call the EMS (EMS-activation group), while some came without activating the EMS (self-transportation group).

Patients were included if they were (1) 14 years and older, (2) with acute stroke and AMI symptoms occurred within 3 days, (3) presenting to the Emergency Department, (4) definitively diagnosed as acute stroke and AMI.

Patients were excluded if they were (1) unwilling to participate in the questionnaire survey, (2) patients with acute stroke or AMI who had started treatment outside of Zhongjiang County and were subsequently transferred to our hospital. (3) the patients were unable to respond to the questionnaires, and no one knew the actual situation.

### Zhongjiang county and EMS setting

**Fig. 1:**
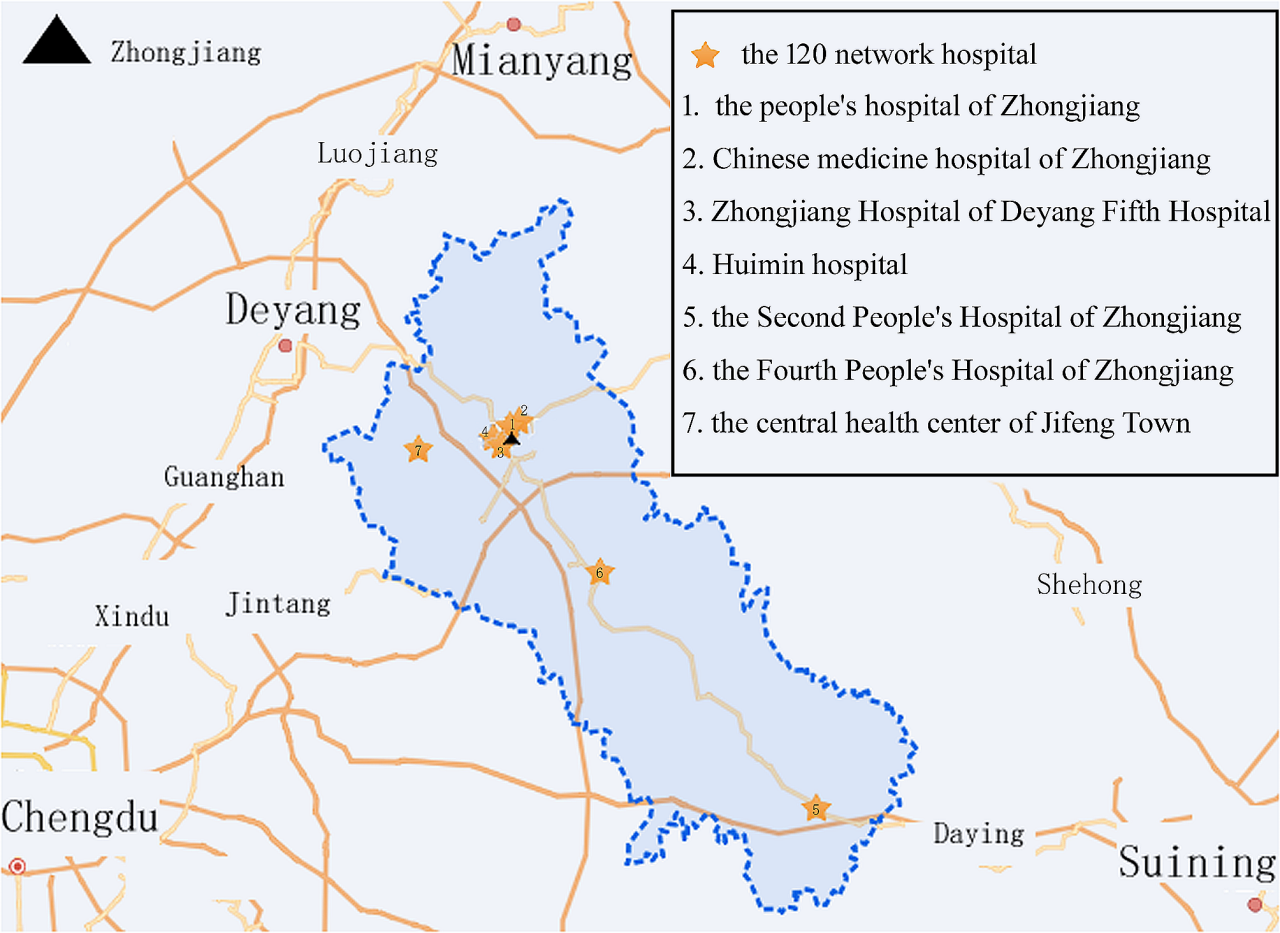
The map of Zhongjiang County and its neighboring areas, and the locations of these 120 network hospitals. The blue dotted line demarcates the boundaries of Zhongjiang County

Zhongjiang County is located in the central Sichuan hilly region, in the northwest of the Sichuan Basin. The county covers an area of 2,200 square kilometers, including 26 towns and 4 townships, totally 522 villages (communities). According to the 2021 census data, Zhongjiang County has a registered population of 1.363 million and a permanent population of 948,000, with an urbanization rate of 42.9% for the permanent population. There are seven hospitals affiliated with the 120 emergency medical network in Zhongjiang County. Figure 1 provides a map of Zhongjiang County and its neighboring areas, highlighting the locations of these 120 network hospitals.

The people’s hospital of Zhongjiang serves as a significant tertiary medical hub in Zhongjiang County, Sichuan Province. It provides round-the-clock services, seven days a week, for intravenous thrombolysis and endovascular intervention for cerebral infarction, as well as PCI-mediated reperfusion therapy for AMI.

### Contents, development, and validation of the questionnaire

The questionnaire comprehensively covers various aspects related to patients’ experiences with acute stroke and AMI, from basic demographic details to their level of disease awareness, healthcare-seeking behavior, and potential causes of delayed EMS activation. Besides, it also explores their hypothetical actions and reasons if a similar situation were to occur again. The specific contents of the questionnaire are detailed in the supplementary document.

The design of the questionnaire initially referenced questionnaires from other studies [6, 12] and was adjusted according to the actual situation. Following this, a pilot testing phase was conducted to evaluate its effectiveness. Based on the feedback of the pilot test and expert reviews, further revisions were made repeatedly to the questionnaire. Finally, the revised questionnaire was deemed ready for larger-scale administration.

### Data collection

Patients who experienced acute stroke and AMI were initially screened upon arrival at the Emergency Department. Subsequently, they or their family member were invited by a trained investigator within 2 days to participate in the study and complete the questionnaire. The data collection was conducted on the phone using a WeChat mini-program, according to the standard data collection protocol developed by the research team.

If the patients were able to express themselves, the patients or their family members would complete the questionnaire based on the patient’s statements. If the patients were unable to respond to the questionnaires due to unconsciousness, aphasia, or being intubated, then their family members would complete the questionnaire based on the actual situation. If the patients were in the Intensive Care Unit or had passed away with no family members present in the hospital, the investigator would complete the questionnaire through a telephone interview. If the family members did not have a phone or were unfamiliar with the process, the investigator would complete the questionnaire or guide them through the process.

The questionnaire items that are most likely to lead to bias are as follows: (1) When inquiring about the symptoms of cardiovascular and cerebrovascular diseases before the onset of the illness, respondents often choose current symptoms. The investigator must clarify that the intent is to gauge understanding of these diseases before onset, not current manifestations. (2) During the inquiry about the distances patients were away from those three places, respondents frequently offer rough estimates in kilometers, which may result in significant errors. For those who are unsure of the distance, the investigator uses navigation software to measure the exact distance. (3) When inquiring about hypothetical actions and reasons in the event of a similar situation recurring, respondents often consider some factors and make choices that may not reflect their actual intentions. Based on their responses during the interview, the investigator will offer guidance to mitigate potential errors.

### Statistical analysis

Categorical variables are presented as frequency (percentage) and were compared using the Chi-square test or Fisher’s exact test. Normally distributed continuous variables are presented as mean ± SD and were compared with the t-test. Non-normally distributed continuous variables are presented as median (95% confidence interval [CI]) and were compared with the Mann–Whitney test if independent or the Wilcoxon test in case of dependent variables. P value < 0.05 was considered statistically signifcant. All data were anonymized and analyzed with SPSS (version 20.0) and R (version 4.2.3).

In our study, Least Absolute Shrinkage and Selection Operator (LASSO) regression analysis was performed to screen the most relevant variables using the glmnet package in R. This popular method avoids overfitting by incorporating the best performance parameters, resulting in a simpler and more easily interpreted model. LASSO regression was chosen for this analysis due to its ability to efficiently perform variable selection and handle multicollinearity. By penalizing the sum of absolute coefficients, LASSO automatically shrinks some coefficients to zero, selecting only the most relevant variables and reducing the influence of less correlated predictors. Furthermore, LASSO’s computational efficiency allows it to handle large datasets effectively. In comparison to other regression methods, LASSO offers a unique combination of variable selection, regularization, and prediction accuracy, making it a robust choice for this analysis.

Subsequently, we conducted a logistic regression analysis to further examine the predictors of EMS activation according the results of LASSO regression. In selecting the final logistic regression model, we incorporated variables that were selected by the LASSO regression procedure and those that demonstrated statistical significance. We comprehensively evaluated the model’s goodness of fit and predictive performance using Nagelkerke’s R² value, the p value and the prediction accuracy across different groups. The Harrell’s C-index was used to quantify and validate the concordance performance of the model. To obtain a relatively corrected estimate of the C-index, the model was subjected to bootstrapping validation with 1,000 bootstrap resamples. Through the multivariable logistic regression analysis, we obtained some results that more accurately depicts the relationship between the predictors and the EMS activation outcome.

## Results

During the study period, 1082 patients with acute stroke and AMI were admitted to our hospital. Among them, 582 patients had acute cerebral infarction, 354 patients had acute cerebral hemorrhage, 48 patients had acute spontaneous subarachnoid hemorrhage, and 98 patients had AMI. However, a total of 153 patients were excluded from the study for various reasons, including unwillingness to participate in the questionnaire survey, failure of the investigator to complete the survey, invalid questionnaires, and patients with acute stroke or AMI who had started treatment outside of Zhongjiang County and were subsequently transferred to our hospital, as they met the exclusion criteria. The final result was that we collected 929 valid questionnaires. Among them, 243 (26.16%) called for EMS (EMS-activation group), while the remaining did not call for EMS (self-transportation group).

### Social demographics, cardiovascular history, risk factors

Table 1 lists social demographics, cardiovascular history, risk factors in the overall population and comparison between EMS-activation group and self-transportation group. No statistical differences were observed in age, gender, educational level, marriage status, risk factors and previous cardiovascular or cerebrovascular diseases. Only the answers regarding whether they have ever received first aid education showed statistical differences between the two groups. The self-transportation group was more likely to report not knowing the 120 emergency phone number (11.4% vs. 6.2% *P* = 0.02), whereas the EMS-activation group was more likely to have studied first aid through media or the Internet (9.5% vs. 5.2% *P* = 0.02).

**Table 1:** Social demographics, cardiovascular history, risk factors in the overall population and comparison between EMS-activation group and self-transportation group

Variable	Overall	EMS activation	Self-transported	*p* Value
Age, years [IQR]	71[61–78]	71[62–80]	71[61–78]	0.49
Sex (male), n (100%)	505(54.4%)	129(53.1%)	376(54.8%)	0.64
Educational level				0.17
primary school	565(60.8%)	136(56.0%)	429(62.5%)	
junior middle school	125(13.5%)	37(15.2%)	88(12.8%)	
senior middle school and above	49(5.3%)	18(7.4%)	31(4.5%)	
I haven’t been to school	190(20.5%)	52(21.4%)	138(20.1%)	
Marriage status				0.08
single(unmarried, divorced, widowed), n (100%)	179(19.3%)	56(23.0%)	123(17.9%)	
married, n (%)	750(80.7%)	187(77.0%)	563(82.1%)	
n (100%)				
diabetes mellitus	175(18.8%)	45(18.5%)	130(19.0%)	0.88
hypertension	600(64.6%)	161(66.3%)	439(64.0%)	0.53
dyslipidemia	42(4.5%)	13(5.3%)	29(4.2%)	0.47
familiar history	44(4.7%)	12(4.9%)	32(4.7%)	0.86
smoking	241(25.9%)	68(28.0%)	173(25.2%)	0.40
no	180(19.4%)	40(16.5%)	140(20.4%)	0.18
Previously suffered from cardiovascular and cerebrovascular diseases, n (100%)	244(26.3%)	64(26.3%)	180(26.2%)	0.98
the question of whether you have ever received first aid education, n (100%)				
no, I didn’t even know the 120 emergency phone number	93(10.0%)	15(6.2%)	78(11.4%)	0.02
I only know that I can call 120 when you need first aid	752(80.9%)	199(81.9%)	553(80.6%)	0.66
I had studied it in my school education courses	51(5.5%)	10(4.1%)	41(6.0%)	0.27
I had studied in the media and on the Internet	59(6.4%)	23(9.5%)	36(5.2%)	0.02

### Understandings of cardiovascular and cerebrovascular diseases

Table 2 lists understandings of cardiovascular and cerebrovascular diseases in the overall population and comparison between EMS-activation group and self-transportation group. The EMS-activation group was more likely to believe that cardiovascular and cerebrovascular diseases have symptoms such as disorders of consciousness or convulsions (11.1% vs. 6.7% *p* = 0.03) and chest pressure (7.8% vs. 3.8% *p* = 0.02). In addition, it is worth noting that 42.1% of them reported that they had no understanding of cardiovascular and cerebrovascular diseases.

**Table 2:** Understandings of diseases in the overall population and comparison between EMS-activation group and self-transportation group (sorted by overall from high to low)

Variable, *n* (100%)	Overall	EMS activation	Self-transported	*p* Value
I have no understanding of cardiovascular and cerebrovascular diseases	391(42.1%)	98(40.3%)	293(42.7%)	0.52
one side of the limb is weak, numb, and clumsy	279(30.0%)	66(27.2%)	213(31.0%)	0.26
poor speaking or difficulty in understanding the language	151(16.3%)	42(17.3%)	109(15.9%)	0.61
one side of the face numbness or askew of the mouth	125(13.5%)	30(12.3%)	95(13.8%)	0.56
headache	109(11.7%)	36(14.8%)	73(10.6%)	0.08
headache associated with nausea and vomiting	101(10.9%)	24(9.9%)	77(11.2%)	0.56
dizziness	84(9.0%)	23(9.5%)	61(8.9%)	0.79
chest pain	81(8.7%)	19(7.8%)	62(9.0%)	0.56
disorders of consciousness or convulsions	73(7.9%)	27(11.1%)	46(6.7%)	0.03
weakness and asthenia	65(7.0%)	19(7.8%)	46(6.7%)	0.56
dyspnea	62(6.7%)	20(8.2%)	42(6.1%)	0.26
vertigo associated with nausea and vomiting	57(6.1%)	18(7.4%)	39(5.7%)	0.34
nausea, vomiting	48(5.2%)	18(7.4%)	30(4.4%)	0.07
chest pressure	45(4.8%)	19(7.8%)	26(3.8%)	0.01
stomach burning	27(2.9%)	8(3.3%)	19(2.8%)	0.67
optic rotation or balance disorder	24(2.6%)	6(2.5%)	18(2.6%)	0.9
other	23(2.5%)	5(2.1%)	18(2.6%)	0.63
sweating	21(2.3%)	8(3.3%)	13(1.9%)	0.21
left arm pain or shake	16(1.7%)	4(1.6%)	12(1.7%)	1.00
loss or blurred vision on either one side or both eyes	16(1.7%)	4(1.6%)	12(1.7%)	1.00
both eyes are fixed and can not rotate	10(1.1%)	3(1.2%)	7(1.0%)	0.73

### Symptom characteristics

Table 3 lists symptom characteristics when the symptoms occurred in the overall population and comparison between the two groups. The most common symptom in patients with acute stroke was that one side of the limb is weak or numb or clumsy, which occurs in 58.0% of cases. Meanwhile, chest pain was the most common symptom occurring in 73.3% of patients with AMI. One side of the limb is weak or numb or clumsy (50.7% vs. 60.7%, *p* < 0.01) was associated with decreased use of ambulance. At the same time, chest pain(78.6% vs. 50.0%, *p* = 0.03) was associated with increased use of ambulance.

**Table 3:** Symptom characteristics when the symptoms occurred in the overall population and comparison between the two groups

Variable, *n* (100%)	Overall	EMS activation	Self-transported	*p* Value
Acute stroke				
one side of the limb is weak, numb, and clumsy	489(58.0%)	115(50.7%)	374(60.7%)	< 0.01
poor speaking or difficulty in understanding the language	319(37.8%)	91(40.1%)	228(37.0%)	0.41
one side of the face numbness or askew of the mouth	192(22.8%)	47(20.7%)	145(23.5%)	0.38
headache	151(17.9%)	47(20.7%)	104(16.9%)	0.20
dizziness	148(17.6%)	35(15.4%)	113(18.3%)	0.32
headache associated with nausea and vomiting	101(12.0%)	46(20.3%)	55(8.9%)	< 0.001
disorders of consciousness or convulsions	91(10.8%)	50(22.0%)	41(6.7%)	< 0.001
vertigo associated with nausea and vomiting	88(10.4%)	24(10.6%)	64(10.4%)	0.94
optic rotation or balance disorder	60(7.1%)	13(5.7%)	47(7.6%)	0.34
loss or blurred vision on either one side or both eyes	15(1.8%)	8(3.5%)	7(1.1%)	0.03
both eyes are fixed and can not rotate	3(0.4%)	2(0.9%)	1(0.2%)	0.18
other	51(6.0%)	20(8.8%)	31(5.0%)	
Acute myocardial infarction				
chest pain	63(73.3%)	8(50.0%)	55(78.6%)	0.03
grade of chest pain				0.09
mild (grade 1/2/3)	11(17.5%)	2(25.0%)	9(16.4%)	
moderate (grade 4/5/6)	22(34.9%)	0(0.0%)	22(40.0%)	
severe (grade 7/8/9/10)	30(47.6%)	6(75.0%)	24(43.6%)	
sweating	34(39.5%)	6(37.5%)	28(40.0%)	0.85
chest pressure	25(29.1%)	5(31.2%)	20(28.6%)	1.00
dyspnea	23(26.7%)	5(31.2%)	18(25.7%)	0.76
nausea, vomiting	14(16.3%)	5(31.2%)	9(12.9%)	0.13
dizziness	10(11.6%)	3(18.8%)	7(10.0%)	0.39
tired	8(9.3%)	2(12.5%)	6(8.6%)	0.64
stomach burning	5(5.8%)	0(0.0%)	5(7.1%)	0.58
left arm pain or shake	3(3.5%)	0(0.0%)	3(4.3%)	1.00

### The situation when the symptoms occurred

Table 4 lists informations regarding the situation when the symptoms occurred in the overall population and comparison between the two groups. The median time of the onset to hospital arrival in EMS-activation group was 2.5 h, whereas in the self-transportation group, the median time was 5.0 h, with *p* < 0.001. Additionally, in EMS-activation group, the median time of the onset to EMS calling was 2.00 h (Interquartile Range [IQR]: 0.67–5.00 h) and the median time of EMS calling to hospital arrival was 0.81 h (IQR: 0.48–1.31 h).

**Table 4:** Informations regarding the situation when the symptoms occurred in the overall population and comparison between the two groups

Variable	Overall	EMS activation	Self-transported	*p* Value
The time of the onset to hospital arrival, hours [IQR]	4.00[1.83–13.46]	2.50[1.37–6.70]	5.00[2.00-18.54]	< 0.001
Diagnosis, n (100%)				< 0.001
acute cerebral infarction	525(56.5%)	107(44.0%)	418(60.9%)	
acute cerebral hemorrhage	289(31.1%)	106(43.6%)	183(26.7%)	
acute spontaneous subarachnoid hemorrhage	29(3.1%)	14(5.8%)	15(2.2%)	
acute myocardial infarction	86(9.3%)	16(6.6%)	70(10.2%)	
Time of symptom onset, n (100%)				< 0.01
1 am–4 am	71 (7.6%)	21 (8.6%)	50 (7.3%)	
5 am–8 am	230 (24.8%)	45 (18.5%)	185 (27.0%)	
9 am–12 pm	242 (26.0%)	70 (28.8%)	172 (25.1%)	
1 pm–4 pm	160 (17.2%)	33 (13.6%)	127 (18.5%)	
5 pm–8 pm	157 (16.9%)	55 (22.6%)	102 (14.9%)	
9 pm–midnight	69 (7.4%)	19 (7.8%)	50 (7.3%)	
When the patient had these symptoms, kilometers [IQR]				
the distance between the patient and the nearest township hospital	2[1–5]	2[1–5]	3[1–5]	0.03
the distance between the patient and the nearest first aid station (the 120 network hospital)	12[4–20]	10[2–18]	14[5–20]	< 0.001
the distance between the patient and the people’s hospital of Zhongjiang	20[6.5–38.5]	15[3–30]	20[10–40]	< 0.001
Where was the patient when these symptoms occur? n (100%)				0.03
home	750(80.7%)	183(75.3%)	567(82.7%)	
public place	76(8.2%)	28(11.5%)	48(7.0%)	
in the work or in the labor	52(5.6%)	14(5.8%)	38(5.5%)	
with relatives or friends	22(2.4%)	5(2.1%)	17(2.5%)	
other	29(3.1%)	13(5.3%)	16(2.3%)	
Who was there when the patient was symptomatic?				
the patient’s wife or husband / patient’s partner	419(45.1%)	99(40.7%)	320(46.6%)	0.11
the patient’s son or daughter	261(28.1%)	60(24.7%)	201(29.3%)	0.17
no one	184(19.8%)	55(22.6%)	129(18.8%)	0.20
friends of the patient	62(6.7%)	17(7.0%)	45(6.6%)	0.82
colleagues of the patient’s work	19(2.0%)	5(2.1%)	14(2.0%)	1.00
others	61(6.6%)	22(9.1%)	39(5.7%)	0.07
Who realized the serious problem before making an emergency call or going to the hospital?				< 0.001
relatives	679(73.1%)	188(77.4%)	491(71.6%)	
the patient	129(13.9%)	15(6.2%)	114(16.6%)	
friend	41(4.4%)	16(6.6%)	25(3.6%)	
no one	29(3.1%)	3(1.2%)	26(3.8%)	
doctors in a private practice	12(1.3%)	5(2.1%)	7(1.0%)	
other	39(4.2%)	16(6.6%)	23(3.3%)	
Where did the patient go in the first time?				0.08
the people’s hospital of Zhongjiang	597(64.3%)	164(67.5%)	433(63.1%)	
nearby township health center	202(21.7%)	46(18.9%)	156(22.7%)	
private practice, individual doctors	42(4.5%)	5(2.1%)	37(5.4%)	
hospital near	32(3.4%)	13(5.3%)	19(2.8%)	
other hospitals with a chest pain center / stroke center	18(1.9%)	4(1.6%)	14(2.0%)	
other	38(4.1%)	11(4.5%)	27(3.9%)	
Who had decided that the patient needed further medical help?				< 0.001
the patient’s son or daughter	489(52.6%)	129(53.1%)	360(52.5%)	
the patient’s wife or husband / patient’s partner	171(18.4%)	44(18.1%)	127(18.5%)	
doctor from a nearby township hospital	92(9.9%)	25(10.3%)	67(9.8%)	
the patient	79(8.5%)	5(2.1%)	74(10.8%)	
friends of the patient	17(1.8%)	9(3.7%)	8(1.2%)	
other doctor	14(1.5%)	4(1.6%)	10(1.5%)	
private clinic	10(1.1%)	1(0.4%)	9(1.3%)	
colleagues of the patient’s work	9(1.0%)	3(1.2%)	6(0.9%)	
other	48(5.2%)	23(9.5%)	25(3.6%)	
If the patient didn’t seek help immediately after symptoms, what caused you to do so?				< 0.001
we sought medical attention immediately after the symptoms appeared and did not wait	549(59.1%)	184(75.7%)	365(53.2%)	
think the symptoms will disappear spontaneously	144(15.5%)	18(7.4%)	126(18.4%)	
think they are not important symptoms	138(14.9%)	16(6.6%)	122(17.8%)	
don’t want to disturb others	24(2.6%)	1(0.4%)	23(3.4%)	
think it’s not a heart or brain problem	16(1.7%)	5(2.1%)	11(1.6%)	
the symptoms are unstable	7(0.8%)	1(0.4%)	6(0.9%)	
I hope to consult the doctor that I know well first	6(0.6%)	1(0.4%)	5(0.7%)	
fear	4(0.4%)	1(0.4%)	3(0.4%)	
previous negative experiences with the hospital	2(0.2%)	0(0.0%)	2(0.3%)	
other	39(4.2%)	16(6.6%)	23(3.4%)	

Patients diagnosed with acute cerebral hemorrhage (43.6% vs. 26.7%) and acute spontaneous subarachnoid hemorrhage (5.8% vs. 2.2%) exhibited significantly higher utilization of ambulances compared to those diagnosed with acute cerebral infarction (44.0% vs. 60.9%) and AMI (6.6% vs. 10.2%), with a statistically significant difference (*p* < 0.001).

The distribution of symptom onset times among patients also had statistically significant implications for their decision to call an ambulance (*p* < 0.01). There were higher numbers of ambulance calls from 5 pm to 8 pm (22.6% vs. 14.9%), 9 pm to midnight (7.8% vs. 7.3%), 1 am to 4 am (8.6% vs. 7.3%), and 9 am to 12 pm (28.8% vs. 25.1%). Conversely, lower numbers of ambulance calls were observed between 5 am and 8 am (18.5% vs. 27.0%), 1 pm and 4 pm (13.6% vs. 18.5%).

When the patient had these symptoms, the median distance to the nearest township hospital was 2 km for the EMS-activation group and 3 km for the self-transportation group (*p* = 0.03). The median distance between the patient and the nearest 120 network hospital was 10 km for the EMS-activation group and 14 km for the self-transportation group (*p* < 0.001). Meanwhile, the median distance between the patient and the people’s hospital of Zhongjiang was 15 km for the EMS-activation group and 20 km for the self-transportation group (*p* < 0.001).

When asked where was the patient when these symptoms occur, patients who were in a public place (11.5% vs. 7.0%) had a higher utilization of ambulance services compared to those who were at home (75.3% vs. 82.7%) (*p* = 0.03).

Before making an emergency call or going to the hospital, the patient’s relatives (77.4% vs. 71.6%), friend (6.6% vs. 3.6%), doctors in a private practice (2.1% vs. 1.0%), and other individual (6.6% vs. 3.3%) who realized that this was a serious problem were associated with increased use of ambulance compared to the patient (6.2% vs. 16.6%), and no one (1.2% vs. 3.8%) (*p* < 0.001). While, when doctor from a nearby township hospital (10.3% vs. 9.8%), other doctor (1.6% vs. 1.5%), the patient’s friends (3.7% vs. 1.2%), colleagues (1.2% vs. 0.9%), and other individual (9.5% vs. 3.6%) who decided that the patient needed further medical help, there were higher numbers of ambulance calls than the patient (2.1% vs. 10.8%), the patient’s partner (18.1% vs. 18.5%), and private clinic (0.4% vs. 1.3%) (*p* < 0.001).

When asked what caused them not to seek help immediately after symptoms appeared, the self-transportation group was more likely to believe that the symptoms would disappear spontaneously (18.4% vs. 7.4%), they were not important symptoms (17.8% vs. 6.6%), didn’t want to disturb others (3.4% vs. 0.4%) compare to the EMS-activation group. While, the EMS-activation group was even more likely to think that it wasn’t a heart or brain problem (2.1% vs. 1.6%) (*p* < 0.001).

### Information of the self-transportation group: how to hospital, reasons, choices and strategies for future situations

Table 5 lists Informations about how patients arrived at the hospital, their reasons for this choice, and their actions if such a situation were to occur again among the 686 patients in the self-transportation group. The results of the survey show that the majority of patients arrive at the hospital by private transportation, either driven by a relative or friend (56.6%) or by the patient themselves (24.2%). Meanwhile, a minority chose to charter a private vehicle (11.5%).

**Table 5:** Informations about how patients arrived at the hospital, their reasons for this choice, and their actions if such a situation were to occur again among those who self-transport

Variable	Overall
How did the patient get to the hospital?, n (100%)	
the private transport driven by the patient’s relative / friend	388(56.6%)
the private transport driven by the patient	166(24.2%)
chartered a private vehicle	79(11.5%)
other	53(7.7%)
Why don’t you call 120?, n (100%)	
I believed that a private transport was much faster	267(38.9%)
I didn’t think this was a health problem serious enough to be called 120	171(24.9%)
I didn’t think of it	92(13.4%)
I was already in a private vehicle	64(9.3%)
I wanted to choose the hospital by myself	20(2.9%)
I didn’t know how to dial 120	11(1.6%)
I didn’t want to disturb others	5(0.7%)
I didn’t think 120 could help me	1(0.1%)
other	55(8.0%)
I believed that a private transport was much faster	267(38.9%)
I didn’t think this was a health problem serious enough to be called 120	171(24.9%)
If the time goes back when the patient first became ill, or if the patient experiences the same symptoms again, will you call 120 or go back to the hospital through private transportation?, n (100%)	
I will call the 120	311(45.3%)
I will still go to the hospital through private transportation	373(54.4%)
other	2(0.3%)
Why again with a private vehicle, n (100%)	
It’s faster to drive by yourself	229(73.2%)
It’s more convenient to drive by yourself	84(26.8%)
Thinking the disease is not serious	40(12.8%)
Driving by yourself is more economical	9(2.9%)
other	11(3.5%)
Why again with calling the 120, n (100%)	
The medical staff in the ambulance are more professional	91(29.1%)
Ambulance is faster	80(25.6%)
Thinking the disease is serious	68(21.7%)
Convenient ambulance	28(8.9%)
other	46(14.7%)

When asked why they didn’t call 120 EMS, the most common reasons were that patients believed a private transport would be faster (38.9%), they didn’t think the health issue was severe enough to require an ambulance (24.9%), some patients didn’t consider it (13.4%), and some were already in a private vehicle (9.3%).

When asked if they would call 120 or use private transportation to return to the hospital in the event that the time were to go back and the patients became ill again or displayed the same symptoms, the results were that 45.3% would call 120, while 54.4% would still choose private transportation to go to the hospital. The main reason for preferring private transportation was that it was faster (73.2%), more convenient (26.8%), or patients thought that the disease wasn’t serious (12.8%). Some also believed that driving themselves was more economical (2.9%). For those who would call 120 again, the top reasons were that the medical staff in the ambulance was more professional (29.1%) and that ambulance was faster (25.6%). Other reasons included thinking the disease was serious (21.7%), believing the ambulance service was more convenient (8.9%).

### Result of Lasso regression analysis

**Fig. 2:**
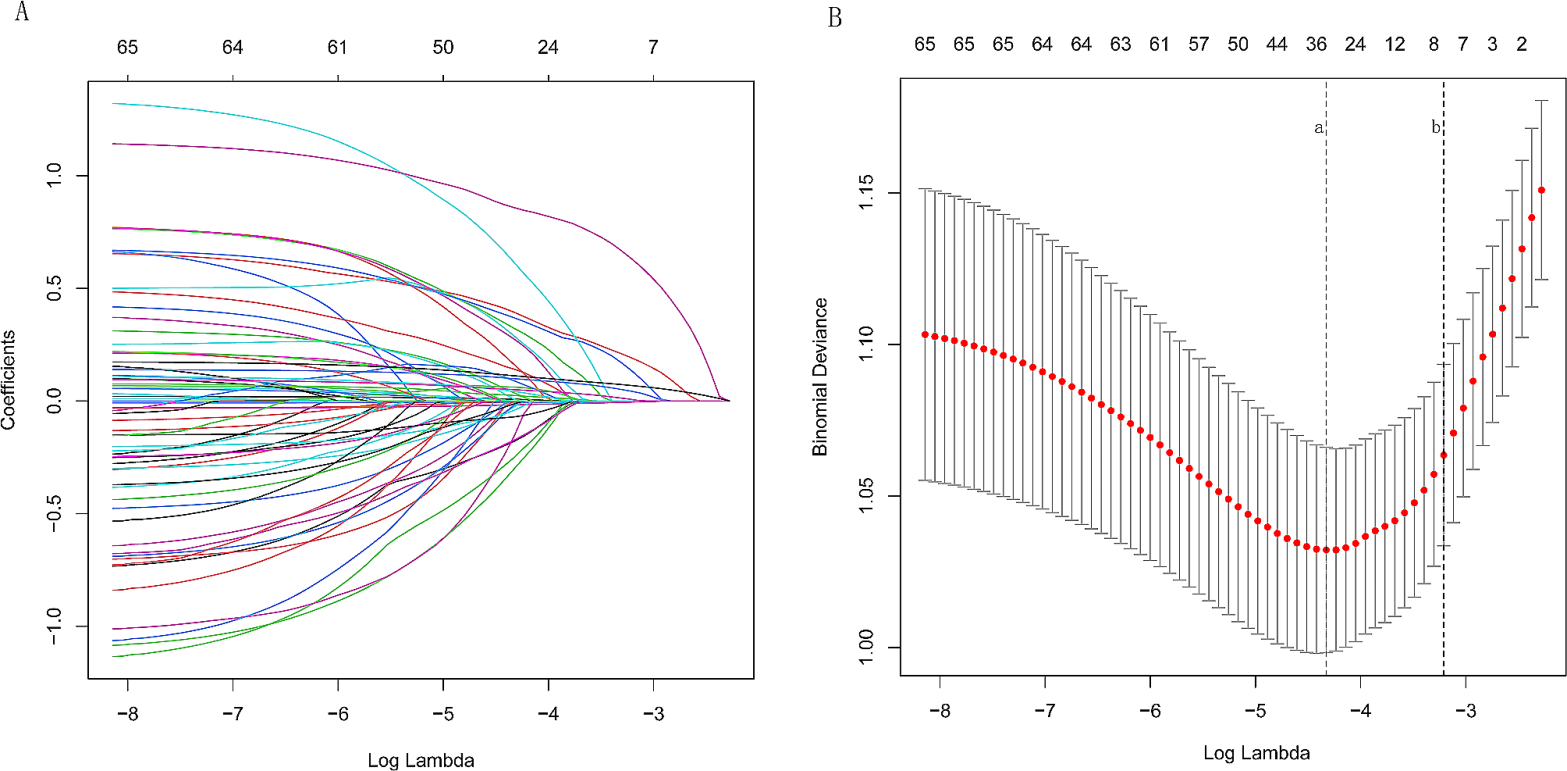
The least absolute shrinkage and selection operator (LASSO) regression analysis. Prognosis-related variates selection in the LASSO regression (**A**). The selection of LASSO regression truncation value (**B**). The dotted line a represents the lambda corresponding to the lowest error mean. The dotted line b represents the maximum lambda corresponding to the error mean within one standard deviation of the minimum

Figure 2 demonstrates the results of LASSO regression analysis, which was performed to screen variates to avoid overfitting and to generate a simpler interpreted model. Finally, 32 prognostic variates were selected, including diagnosis, the distance between the patient and the nearest 120 network hospital, the distance between the patient and the people’s hospital of Zhongjiang, the question of whether you have ever received first aid education (I didn’t even know the 120 emergency phone number, I had studied it in my compulsory education courses, I had studied in the media and on the Internet), the understanding of the symptoms of cardiovascular and cerebrovascular diseases prior to this illness (chest pressure, headache, headache associated with nausea and vomiting, one side of the face numbness or askew of the mouth, both eyes are fixed and can not rotate, disorders of consciousness or convulsions, having no understanding of cardiovascular and cerebrovascular diseases), the symptoms experienced by the patient with AMI (chest pressure, sweating, nausea and vomiting), grades of chest pain, the symptoms experienced by the patient with acute stroke(headache, headache associated with nausea and vomiting, dizziness, one side of the limb is weak or numb or clumsy, loss or blurred vision on either one side or both eyes, optic rotation or balance disorder, disorders of consciousness or convulsions), the place where the patient was when these symptoms occur, the person who was there when the patient was symptomatic(no one, the patient’s son or daughter), the person who realized that the problem was serious before making an emergency call or going to the hospital, the place where the patient sought medical attention in the first instance, the person who had decided that the patient needed further medical help, the reasons that caused the patient didn’t seek help immediately after symptoms.

### Result of multivariable logistic regression analysis

Table 6 lists the final result of multivariable logistic regression analysis. It showed that diagnosed as AMI (OR 0.22, 95%CI 0.06 to 0.88) or acute cerebral infarction (OR 0.26, 0.10 to 0.68), the distance between the patient and the nearest 120 network hospital when the patient had these symptoms (OR 0.97, 0.94 to 0.99), the patient believed that the symptoms of cardiovascular and cerebrovascular diseases included headache accompanied by nausea and vomiting(OR 0.35, 0.18 to 0.69), one side of the face numbness or askew of the mouth (OR 0.51, 0.28 to 0.91) before the onset of this illness, the patient’s son or daughter was there when the patient was symptomatic (OR 0.58, 0.37 to 0.94), the patient (OR 0.19, 0.05 to 0.72) and the patient’s partner (wife or husband) (OR 0.36, 0.16 to 0.85) had decided that the patient needed further medical help, Among patients who did not seek immediate help after symptom onset, thinking that the symptoms will disappear spontaneously (OR 0.34, 0.13 to 0.92) or not wanting to disturb others (OR 0.06, 0.01 to 0.66) or believing that they are not important symptoms (OR 0.15, 0.05 to 0.42) were factors independently associated with less ambulance use. Age (OR 1.02, 1.00 to 1.04), and stroke patients have experienced symptoms of disturbance of consciousness or convulsions (OR 2.99, 1.72 to 5.2) were independent factors associated with increased ambulance use. The model is statistically significant with a p-value less than 0.001, and its Nagelkerke’s R^2^ is 0.34. Meanwhile, the model’s overall comprehensive prediction accuracy is 80.0% (for the self-transportation group, the prediction accuracy is 93.9%, while, for the EMS-activation group is 40.7%). The C-index for the model was 0.812 (95% CI: 0.810–0.814), and upon bootstrapping validation, it was validated to be 0.744, indicating moderate accuracy of the model.

**Table 6:** Independent factors associated with choosing or not choosing ambulance in patients with acute stroke and acute myocardial infarction

OR	OR	95%CI	*p* Value
Diagnosis, acute myocardial infarction	0.22	0.06 to 0.88	0.03
Diagnosis, acute cerebral infarction	0.26	0.10 to 0.68	0.01
When the patient had these symptoms, the distance between the patient and the nearest first aid station	0.97	0.94 to 0.99	0.01
Age	1.02	1.00 to 1.04	0.03
Before the onset of this illness, the patient believed that the symptoms of cardiovascular and cerebrovascular diseases included headache accompanied by nausea and vomiting.	0.35	0.18 to 0.69	< 0.01
Before the onset of this illness, the patient believed that the symptoms of cardiovascular and cerebrovascular diseases included one side of the face numbness or askew of the mouth	0.51	0.28 to 0.91	0.02
Stroke patients have experienced symptoms of disturbance of consciousness or convulsions	2.99	1.72 to 5.2	< 0.001
Who was there when the patient was symptomatic? the patient’s son or daughter	0.58	0.37 to 0.94	0.03
Who had decided that the patient needed further medical help?			
the patient	0.19	0.05 to 0.72	0.01
the patient’s wife or husband / patient’s partner	0.36	0.16 to 0.85	0.02
If the patient didn’t seek help immediately after symptoms, what caused you to do so?			
think the symptoms will disappear spontaneously	0.34	0.13 to 0.92	0.03
don’t want to disturb others	0.06	0.01 to 0.66	0.02
think they are not important symptoms	0.15	0.05 to 0.42	< 0.001

## Discussion

There is still ambulance underusage among patients with acute stroke and AMI in county territory of China. In our study, 26.16% of the patients required the services of EMS, which is comparable to the findings of a study conducted seven years ago by Qilu Hospital of Shandong University, where 21.6% of patients with acute coronary syndrome (ACS) were transported to the hospital via ambulance [6]. However, in Northern Italy, 65.2% of patients with ST-segment elevation acute coronary syndromes called for EMS [12]. Meanwhile, in Ireland, the percentage of ACS patients using ambulances was 27% [8] or 40.1% [10]. Therefore, the proportion of our patients with acute stroke and AMI who call the 120 still requires enhancement.

It is worth noting that 80.9% of individuals only know that they can call the 120 when they need first aid, and they have not received any formal first aid education. In addition, 10% of individuals are unaware of the 120 emergency phone number altogether. This also shows that the level of first aid education in China remains low [13]. Furthermore, the EMS-activation group tended to have a higher likelihood of having studied first aid through media or the Internet. In addition, 40.9% of overall patients didn’t seek help immediately after symptoms appeared. When asked what caused this, 15.5% of overall patients believed that the symptoms would disappear spontaneously, while 14.9% thought that they were not important symptoms. These revelations highlight the importance of not only promoting awareness of EMS but also enhancing public education on common health issues related to cardiovascular and cerebrovascular diseases. Such efforts would contribute significantly to improving the level of emergency first aid for acute stroke and heart attacks.

To enhance first aid education, there are multifaceted approaches encompassing mandatory school programs, community workshops, mass media campaigns, and partnerships with non-governmental organizations and private sectors to ensure widespread knowledge and skills.

Moreover, due to the widespread ownership of cars currently, many individuals opt to drive themselves, relatives, or friends to the hospital. The most common reasons for not calling the EMS were patients’ belief that private transportation would be swifter (38.9%) and their assessment that the health issue was not severe enough to necessitate an ambulance (24.9%). In addition, among those in the self-transportation group, 54.4% would still opt for private transportation if the situation arose again, citing the primary reasons for believing that the private vehicle was faster (73.2%), more convenient (26.8%), and the disease wasn’t serious (12.8%). On the other hand, those who would call the 120 in similar circumstances primarily cite the ambulance’s professional medical staff (29.1%), its speed (25.6%), and the seriousness of the illness (21.7%) as reasons for their choice. Correspondently, in Italy, Among those who did not activate EMS, 45.5% believed their symptoms were unrelated to a significant health issue, and 34.7% thought the private vehicle would be faster than an ambulance [12]. In summary, our stroke/chest pain center must diligently strive to enhance public awareness regarding acute stroke and heart attacks, and to popularize first aid education.

On the other hand, when patients presented these symptoms, the EMS-activation group had a median distance of 2 km to the nearest township hospital, 12 km to the nearest 120 network hospital, and 20 km to the people’s hospital of Zhongjiang. Notably, the EMS-activation group tended to have shorter distances to these three locations compared to the self-transportation group. Based on this, we propose that all township hospitals should be integrated into the 120 emergency networks and equipped with emergency first aid capabilities, as well as pre-hospital care and transportation abilities. Furthermore, the implementation strategies for this integration involve Securing government backing, obtaining administrative directives, establishing a coordination mechanism, standardizing protocols, upgrading infrastructure and equipment, training staff, and establishing communication links, among other steps. This integration is expected to increase efficiency in emergency response, improve resource utilization, enhance patient experience, and strengthen public trust in the healthcare system, among other benefits.

Patients were more inclined to opt for an ambulance when symptoms occurred at night, this is similar to another study on ACS [6]. When symptoms occurred, 80.7% of patients were at home, exhibiting a lower utilization of EMS compared to those who were in public places. Additionally, 45.1% of them were accompanied by their partner, and 28.1% were with their son or daughter. Notably, those accompanied by family members also tended to have a lower utilization of EMS. This might be caused by the perception that it wasn’t a serious problem. Prior research has demonstrated that patients tend to call an ambulance when they perceive their symptoms as being sufficiently serious [6, 11]. Furthermore, in our study, before making an emergency call or going to the hospital, the patient and no one who realized that this was a serious problem were associated with decreased use of ambulance compared to when the patient’s relatives, friends, or doctors in private practice were involved. While, when the patient, the patient’s partner, and the private clinic decided that the patient needed further medical help, there were lower numbers of ambulance calls. These further indicate that patients, their families, and private clinic doctors lack adequate understanding of the potentially grave consequences of acute stroke and AMI, as well as the critical importance of prompt medical intervention.

Finally, in discussing our findings, we must address the potential issue of selection bias and its impact on the generalizability of our results. Our samples originated from a central hospital in Zhongjiang County, and neighboring hospitals may have also included minor patients. Selection bias may also arise due to voluntary participation, a unique patient population, and investigator variations. Given that there are over 2,000 county units in China, the specific socioeconomic, demographic, and healthcare context of Zhongjiang County may limit the generalizability of our findings. However, we believe that many of our findings can still provide valuable insights for many other regions with similar characteristics. We recommend improved training for investigators and enhanced research methods to mitigate bias and improve the reliability and generalizability of future studies.

## Conclusions

In summary, Ambulance underutilization persists among patients with acute stroke and AMI in county territory of China, coupled with low levels of first aid education. Our findings underscore that it is needed to raise the level of first aid education and awareness about EMS. Moreover, it is crucial for private clinic doctors and the public to recognize typical symptoms and potential dangers of acute stroke and AMI, as well as the significance of prompt EMS services and timely treatment at chest pain/stroke centers. Finally, we propose that all township hospitals should be integrated into the 120 emergency networks and equipped with emergency first aid capabilities, pre-hospital care, and transportation abilities. These efforts would contribute significantly to improving the level of emergency first aid for acute stroke and heart attacks.

### Electronic supplementary material

Below is the link to the electronic supplementary material.

Supplementary Material 1

## Data Availability

The datasets that were used and/or analyzed during the current study are available from the corresponding author upon reasonable request.

## Abbreviations

EMS: Emergency Medical Service
AMI: Acute Myocardial Infarction
LASSO: Least Absolute Shrinkage and Selection Operator
ACS: Acute Coronary Syndrome
PCI: Percutaneous Coronary Intervention
IQR: Interquartile Range
