# Supplementary material for: Analysis of barriers associated with emergency medical service activation in patients with acute stroke and acute myocardial infarction from Zhongjiang County of Sichuan Province in China
Source: BMC Emerg Med. 2024 Jul 9;24:113. doi: 10.1186/s12873-024-01035-5 (PMC11234688; doi:10.1186/s12873-024-01035-5)
Supplement: Supplementary file 1 — Supplementary Material 1 [file 12873_2024_1035_MOESM1_ESM.docx]

**Informed consent: The early diagnosis and treatment of patients with acute ischemic stroke and acute myocardial infarction is crucial. Often, these patients fail to activate the emergency medical service system (EMSS) in a timely manner, due to various reasons, which can lead to delayed treatment and a worse prognosis for the corresponding diseases. Participating in this questionnaire survey will help us analyze the reasons for this delay and target our efforts to address these issues. This will enable us to better manage the care of patients with acute stroke and acute myocardial infarction, improving outcomes and providing more benefits to related patients. Please be advised that your decision to participate in this survey will not impact your medical care.**

**Do you agree to participate in this questionnaire survey: Yes** □  **No** □

Time to fill in the questionnaire:  year month date ____:____

Age：________ Gender male □ female □

Patients fill in □ / patient family fill in □ / investigators fill in □

1. Date and time of symptom onset: year month date ____:____

1. The time of calling 120 and hospital arrival(if not called)：

year month date ____:____

1. Educational level of the patients

□ primary school

□ middle school

□senior middle school

□university and above

□ I haven't been to school

1. Marriage status of the patient:

□single

□married

□divorced

□widowed

□other______________

1. Risk factors for cardiovascular and cerebrovascular diseases (more than one item) [Multiple choice]

□ diabetes mellitus

□hypertension

□dyslipidemia

□familiar history

□smoking

□no

1. Has the patient ever had cardiovascular and cerebrovascular diseases?

□yes

□no

1. Have you ever received any first-aid education?

□ No, I didn't even know the 120 emergency phone number

□ I only know that I can call 120 when you need first aid

□ I had studied it in my compulsory education courses

□ I had studied in my college courses

□ I Had studied in the media and on the Internet

□ other______________________________________________________

1. What do you think are the symptoms of cardiovascular and cerebrovascular diseases before this illness?[multiple choice]

□chest pain

□stomach burning

□left arm pain/shake

□chest pressure

□dyspnea

□weakness and asthenia

□sweating

□nausea, vomiting

□headache

□headache associated with nausea and vomiting

□ dizziness

□ vertigo associated with nausea and vomiting

□ one side of the limb is weak, numb, and clumsy

□one side of the face numbness or askew of the mouth

□poor speaking or difficulty in understanding the language

□both eyes are fixed and can not rotate

□loss or blurred vision on either one side or both eyes

□optic rotation or balance disorder

□disorders of consciousness or convulsions

□other______________________________________________________

□I have no understanding of cardiovascular and cerebrovascular diseases

1. (Acute myocardial infarction patients fill in) What of the following discomfort or symptoms did the patient experience?(Can more than one) [Multiple choice]

□ chest pain

□stomach burning

□left arm pain/shake

□chest pressure

□dyspnea

□ tiredness

□sweating

□nausea, vomiting

□ dizziness

□other______________________________________________________

1. (Acute myocardial infarction patients fill in) From Grade 1 (the weakest pain you have ever experienced) to 10 (the most severe pain you have ever experienced), how many symptoms are the patient's symptoms?_____
2. (Acute stroke patients fill in) What of the following discomfort or symptoms did the patients experience?(Can more than one) [Multiple choice]]]

□headache

□headache associated with nausea and vomiting

□ dizziness

□ vertigo associated with nausea and vomiting

□ one side of the limb is weak, numb, and clumsy

□one side of the face numbness or askew of the mouth

□poor speaking or difficulty in understanding the language

□both eyes are fixed and can not rotate

□loss or blurred vision on either one side or both eyes

□disorders of consciousness or convulsions

□optic rotation or balance disorder

□other______________________________________________________

1. Was that the first time that the patient had experienced these symptoms?

□yes □no

1. When the patient developed these symptoms, where the specific location of the patient was located ____________________________ (Specific to * *village or * * street)); At that time, how many kilometers was the patient away from the nearest township health center?__________, How many kilometers away from Zhongjiang County People's Hospital __________; In addition to the people's hospital of Zhongjiang, the patient from the following which first aid station nearest, about how many kilometers?

□Chinese medicine hospital of Zhongjiang __________

□Zhongjiang Hospital of Deyang Fifth Hospital__________

□Huimin hospital of integrated traditional chinese and western medicine __________

□the Second People's Hospital of Zhongjiang County __________

□the Fourth People's Hospital of Zhongjiang County__________

□the central health center of Jifeng Town__________

□do not know where to be the nearest and approximate distance

□it is the closest to the people's hospital of Zhongjiang

1. Where was the patient when these symptoms occur?

□home

□in the work or in the labor

□with relatives / friends

□public place

□in the vehicle

□stadium

□other__________________________

1. Who was there when the patient was symptomatic?(Can more than one) [Multiple choice]

□no one

□the patient's wife or husband / patient's partner

□the patient's son or daughter

□colleagues of the patient's work

□friends of the patient

□ others __________

1. Who realized that the problem was serious before making an emergency call or going to the hospital?

□no one

□the patient

□relatives

□friend

□doctors in a private practice

□pharmacy staff

□other doctor

□other_______________________

1. Where did the patient seek medical attention in the first instance?

□private practice, individual doctors

□drugstore

□nearby township health center

□hospital near

□the people's hospital of Zhongjiang

□other hospitals with a chest pain center / stroke center

□other ______________________

1. Who had decided that the patient needed further medical help?

□the patient

□the patient's wife or husband / patient's partner

□the patient's son or daughter

□colleagues of the patient's work

□friends of the patient

□doctor from a nearby township hospital

□private clinic

□drugstore

□other doctor

□other_________________________

1. If the patient didn't seek help immediately after symptoms, what caused you to do so?

□think the symptoms will disappear spontaneously

□don't want to disturb others

□think they are not important symptoms

□think it's not a heart or brain problem

□the symptoms are unstable

□previous negative experiences with the hospital

□I hope to consult the doctor that I know well first

□fear

□awkward

□other_________________________

□we sought medical attention immediately after the symptoms appeared and did not wait

1. How did the patient arrive at the people's hospital of Zhongjiang?

□Call 120

□use of private transportation

□township health center ambulance

□other_________________________

The following survey was only available to those presenting to the ED by private transport

1. How did the patient get to the hospital?

□the private transport driven by the patient

□the private transport driven by the patient's relative / friend

□chartered a private vehicle

□other_________________________

1. Why didn't you call 120?

□I didn't think of it

□I didn't think this was a health problem serious enough to be called 120

□I believed that a private transport was much faster

□the ambulance alert violated my privacy

□I wanted to choose the hospital by myself

□negative personal, family, or friend experiences

□I didn't know how to dial 120

□I was already in a private transport

□I didn't want to disturb others

□I didn't think 120 could help me

□other_______________________________________________________

1. If the time goes back when the patient first became ill, or if the patient experiences the same symptoms again, will you call 120 or go back to the hospital through private transportation?

□I will call the 120

□I will still go to the hospital through private transportation

1. About Question 23: Why?

______________________________________________________________________________________________________________________________________________________________________________________________________________________________
